# P240: Necrotising enterocolitis in preterm infants: epidemiology and antibiotic consumption in the Polish neonatology surveillance network neonatal intensive care units in 2009

**DOI:** 10.1186/2047-2994-2-S1-P240

**Published:** 2013-06-20

**Authors:** J Wojkowska-Mach, A Różańska, PB Heczko

**Affiliations:** 1 Chair of Microbiology, Jagiellonian University Medical School, Kraków, Poland; 2 Chair of Microbiology, Jagiellonian University Medical College, Kraków, Poland

## Introduction

Necrotising enterocolitis (NEC) is one of the most unpredictable and devastating diseases in premature infants. It is a big and costly problem especially for infants who are born with very low birth weight (VLBW).

## Objectives

The aim of this study was descriptive epidemiology of NEC in a representative group of very low birth weight Polish newborns.

## Methods

The study covered 910 children with VLBW hospitalized in six neonatal intensive care units in Poland. Case patients were defined according to NeoKISS as VLBW neonates who had clinical symptoms of necrotising enterocolitis (NEC).

Data on potential risk factors which occurred during pregnancy and hospitalization in NICUs, results of microbiological tests and antibiotic treatment were gathered in the year 2009.

Two kinds of indicators were used for the description of antibiotic usage: the duration of treatment (days of treatment, DOTs) expressed in days and the defined daily dose (DDD), according to the ATC/DDD system.

## Results

NEC incidence was 8.7% (12.7% of all cases of infections) and mortality rate was 19%. The risk of NEC development did not differ between NICUs. Chorioamnionitis, late gestational age and low birth weights was identified as a risk factor of NEC.

Catheterization, mechanical ventillation, antibiotic use and other selected procedures were used for a significantly longer period of time in newborns with NEC than in remaining neonates.

2.9 DDDs of antibiotics were used for treatment of NEC and total treatment duration was 1,437 days; the average use of drugs in this group amounted to 0.47 DDD or 23.18 days per case. Level of antbiotic usage was analyzed with correlation to microbiological tests performed and it was non-significantly greater in the group of children with NEC in whom the tests were done.

## Conclusion

A high risk of developing NEC was closely associated with such factors as: a very low infant birth weight, inflammation of the amnion during labour and the use of invasive procedures. No relationship between the consumption of antibiotics or sepsis accompanying NEC or gut colonization with pathogens was observed.

## Disclosure of interest

None declared.

